# Anchored FRET sensors detect local caspase activation prior to neuronal degeneration

**DOI:** 10.1186/1750-1326-6-35

**Published:** 2011-05-23

**Authors:** Ricardo A Figueroa, Veronica Ramberg, Tom Gatsinzi, Malin Samuelsson, Mu Zhang, Kerstin Iverfeldt, Einar Hallberg

**Affiliations:** 1 Department of Neurochemistry, Stockholm University, SE-106 91 Stockholm, Sweden; 2 Department of Biochemistry and Biophysics, Stockholm University, SE-106 91 Stockholm, Sweden

**Keywords:** Amyloid-β, Caspases, FRET, Live Cell Imaging, Neurite degeneration, Neurodegeneration, Spatiotemporal analysis

## Abstract

**Background:**

Recent studies indicate local caspase activation in dendrites or axons during development and in neurodegenerative disorders such as Alzheimer's disease (AD). Emerging evidences point to soluble oligomeric amyloid-β peptide as a causative agent in AD.

**Results:**

Here we describe the design of fluorescence resonance energy transfer (FRET)-based caspase sensors, fused to the microtubule associated protein tau. Specific caspase sensors preferentially cleaved by caspase-3, -6 or -9 were expressed in differentiated human neuroblastoma SH-SY5Y cells. The anchoring of the sensors resulted in high FRET signals both in extended neurites and soma and made analysis of spatiotemporal signal propagation possible. Caspase activation was detected as loss of FRET after exposure to different stimuli. Interestingly, after staurosporine treatment caspase-6 activation was significantly delayed in neurites compared to cell bodies. In addition, we show that exposure to oligomer-enriched amyloid-β peptide resulted in loss of FRET in cells expressing sensors for caspase-3 and -6, but not -9, in both soma and neurites before neurite degeneration was observed.

**Conclusions:**

Taken together, the results show that by using anchored FRET sensors it is possible to detect stimuli-dependent differential activation of caspases and to distinguish local from global caspase activation in live neuronal cells. Furthermore, in these cells oligomer-enriched amyloid-β peptide induces a global, rather than local activation of caspase-3 and -6, which subsequently leads to neuronal cell death.

## Introduction

In comparison with current knowledge about axon growth and guidance, relatively little is known about the intracellular mechanisms of neurite (axon or dendrite) elimination and its relation to apoptosis is still not clear. It has been suggested that axonal degeneration may occur through a local self-destructive program, which is distinct from the proteolytic program that mediates apoptosis [1]. On the other hand removal of dendrites from sensory neurons during pruning in *Drosophila melanogaster *is directed by local caspase-like activity [2]. In addition, there is evidence for the involvement of the non-classical executioner caspase-6 in local axonal degeneration induced by trophic deprivation of cultured primary sensory neurons [3]. In these neurons, localization of inactive procaspase-6 was observed in both the axons and cell bodies, whereas procaspase-3 was primarily detected in cell bodies [3]. Caspase-6 activation, as detected by immunostaining of active caspase-6, has been reported to occur in hippocampus and cerebral cortex of mild, moderate, severe and very severe sporadic Alzheimer's disease (AD) [4]. In human ischemia caspase-6 translocates to the nuclei [5] where it plays a role in nuclear lamin cleavage and subsequent chromatin condensation [6]. In contrast, in AD brains immunostaining of active caspase-6 was concentrated in neurites [5], indicating a local effect in neurites.

In tubular cell structures, such as axons and dendrites, propagation of diffusion-mediated signaling is slower compared to in the cytosol of a spherical cell. Local caspase activation may not necessarily lead to a global activation of apoptotic processes in a cell (*cf*. [7]). It is also possible that different parts of a cell, such as nerve terminals, axons or dendrites, are more vulnerable to a particular type of toxic insult and that an apoptotic process is initiated in a specific location followed by spreading of the signal to the soma resulting in apoptotic nerve cell death. Although there are many reliable assays for apoptosis, they generally only distinguish between cells that are dead or alive. The incoherent picture of degenerative processes described in the literature calls for techniques that allow monitoring of apoptotic progression in greater detail.

To investigate early degenerative events in neurites and cell bodies we have developed the first model system for spatiotemporal monitoring of caspase activation in neuronal cells. Caspase-3, -6, and -9 have been implicated both during development of the nervous system and in neurodegenerative disorders, such as AD. Hence, fluorescence resonance energy transfer (FRET)-based sensors preferentially cleaved by caspase-3, -6 and -9 were designed. The sensors localize to microtubules and are therefore highly enriched in axons/neurites. Here, we show that our caspase sensors can in fact be used in live cell imaging of neurite degeneration and cellular apoptosis in an *in vitro *model using differentiated human neuroblastoma SH-SY5Y cells. Our experimental system enables monitoring of local activation of caspases and propagation of the signal in neurites and was used to study caspase activation induced by staurosporine, local oxidative stress and by the AD-associated amyloid β (Aβ) peptide.

## Results

### Design of microtubule associated molecular FRET sensors of activation of specific caspases

**Figure 1:**
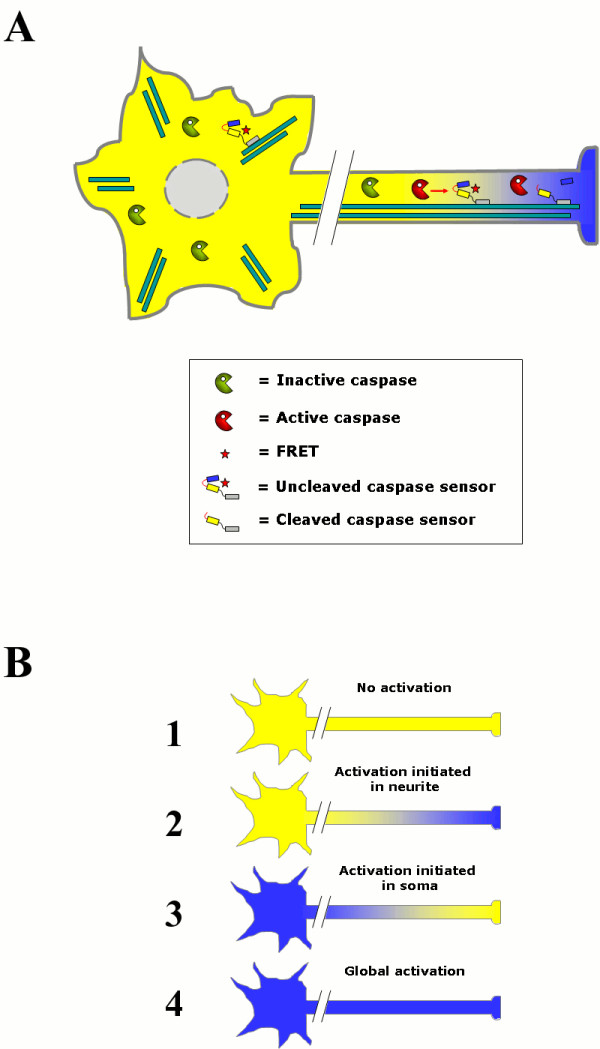
**Schematic illustration of local or global caspase activation**. (A) Microtubule-anchored FRET sensors for spatiotemporal detection of caspase activation in live cells. (B) Different scenarios (1-4) after apoptotic stimuli of differentiated neuronal cells expressing a microtubule-anchored FRET sensor for a specific caspase (in this study: caspase-3, -6, or -9).

**Figure 2:**
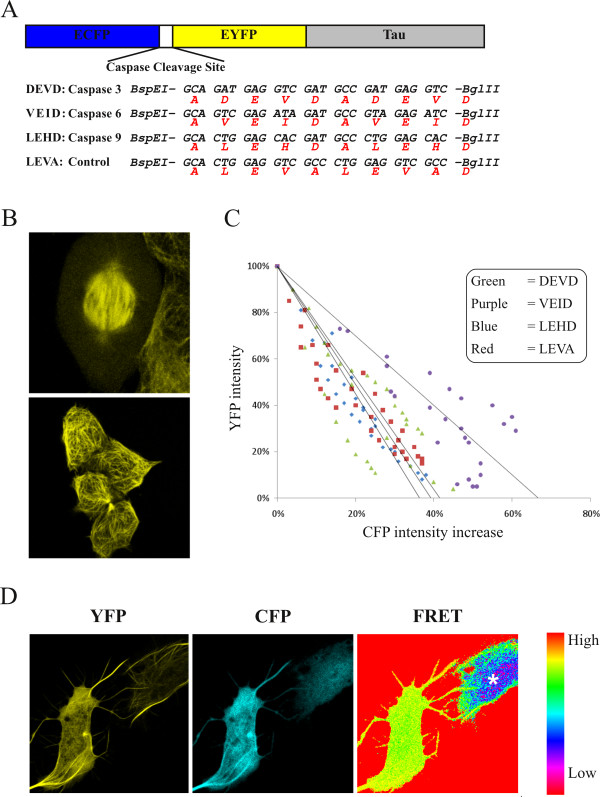
**Design of FRET sensor molecules for detection of local activation of specific caspases**. (**A**) Schematic outline of FRET sensor molecules. ECFP and EYFP are connected with different spacer sequences containing two tandemly arranged tetra-amino acid cleavage sequences preferentially cleaved by different caspases (as specified) or a non-cleavable control tetra-amino acid LEVA sequence. The microtubule binding Tau protein is fused to the C-terminus of EYFP to target the sensors to microtubules. (**B**) Distribution of the caspase-3 (DEVD) sensor to microtubules of mitotic (left) and interphase (right) HeLa cells. (**C**) Quantitative acceptor bleaching experiments in differentiated neuroblastoma SH-SY5Y cells expressing different caspase sensors, showing approximately 50% increase in ECFP fluorescence after bleaching of EYFP. (**D**) U2OS cells expressing FRET sensors for caspase-6 (VEID) treated with 10 μM staurosporine. The apoptotic cell (star) showed maintained localization of EYFP fluorescence to microtubules (right cell), in contrast to ECFP fluorescence, after loss of FRET.

We have constructed cDNA encoding a series of sensor molecules designed to detect activation of specific caspase activities in neurites by a decrease in FRET (Figure 1). The sensors (Figure 2A) are composed of two fluorophores, enhanced cyan fluorescent protein (ECFP) and enhanced yellow fluorescent protein (EYFP), that are separated by a spacer containing two tandemly arranged tetrapeptide sequences preferentially recognized and cleaved by different caspases: DEVD, caspase-3; VEID, caspase-6; LEHD, caspase-9 (*cf. *[8,9]). The tetrapeptide LEVA is resistant to caspase cleavage and serves as a control. In order to localize the caspase sensors to microtubules, which are abundant in axons of neurons, they were fused to the microtubule-associated protein tau. HeLa cells were transiently transfected with plasmids encoding the caspase sensors and their localization was studied by imaging EYFP in live cells using confocal laser scanning microscopy (CLSM). The caspase sensors clearly decorated a filamentous network, characteristic of microtubules in mitosis and interphase (Figure 2B). Overexpression of the sensors did not result in any detectable toxicity and both HeLa and SH-SY5Y cells were morphologically intact for at least 3 days (not shown). Noteworthy, results from a recent *in vivo *study have also shown that neither tau deletion nor overexpression in mice affects axonal transport in retinal ganglion cells [10]. To determine the efficiency of FRET a quantitative acceptor bleach analysis was performed (Figure 2C). All sensors displayed a FRET efficiency of approximately 40 - 60%, a level of intensity that is readily detectable via ratiometric imaging of FRET/ECFP. Thus, the sensor molecules gave strong and stable FRET signals prior to induction of apoptosis and caspase activation. Moreover, staurosporine treated U2OS cells expressing the caspase-6 (VEID) sensor, clearly showed maintained localization of EYFP fluorescence to microtubules after loss of FRET (Figure 2D). In contrast, ECFP fluorescence displayed a diffuse distribution in the cell. This clearly demonstrates that loss of FRET is a consequence of VEID-specific cleavage and is not due to any other proteolytic event.

### Loss of FRET and proteolytic cleavage of sensor molecules in differentiated neuroblastoma cells undergoing apoptosis

**Figure 3:**
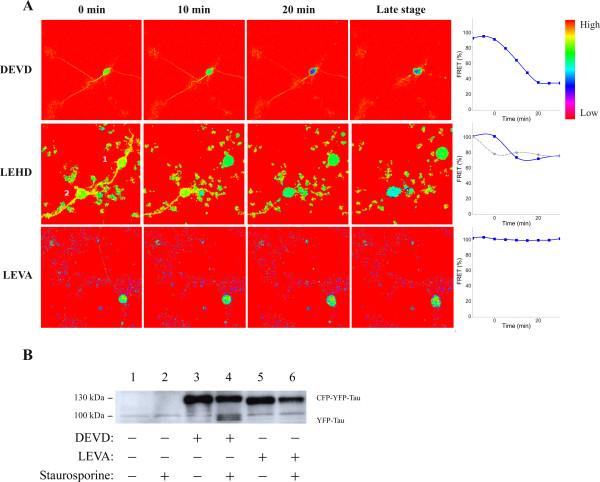
**Monitoring of FRET sensor molecule cleavage in neuronal cells undergoing apopotosis**. Transfected and differentiated SH-SY5Y cells were treated with 10 μM staurosporine to induce apoptosis. (**A**) FRET intensity time-lapse imaging of FRET sensors. Time is indicated on top. The frame before the initial drop in FRET intensity was chosen as zero time point for the caspase-3 (DEVD) and -9 (LEHD) sensors, whereas initiation of neurite retraction was chosen as zero time point for the control (LEVA) sensor. Corresponding graphs show the quantitative change in FRET intensity. The FRET signal decreased in cells expressing the caspase-3 (DEVD), or -9 (LEHD) sensors, but not in cells expressing the control (LEVA) sensor. The gray dashed curve corresponds to the right cell (#1), which has a different zero time point than the left cell (#2) in the LEHD time-lapse experiment. (**B**) Western blot analysis using anti-GFP antibodies of FRET sensor molecules in extracts of cells expressing the caspase-3 (DEVD) sensor or the uncleavable control (LEVA) sensor incubated for 3 hours in the absence or presence of 10 μM staurosporine.

Differentiated human neuroblastoma SH-SY5Y cells were transfected with plasmids encoding the different FRET sensor molecules. The experiments were started by addition of staurosporine, one of the best inducers of apoptosis in many different cell types, after which the cells were imaged by FRET microscopy for 8 hours in order to monitor changes in FRET. In cells expressing sensors for caspase-3 (DEVD) a drop in FRET was detectable before retraction of neurites and the FRET signal continued to decrease for 20 min (Figure 3A). The decrease in FRET signal from the caspase-9 (LEHD) sensor was completed in 10 min (Figure 3A). Cells expressing the control FRET sensor, containing the engineered non-cleavable tetra-amino acid motif (LEVA), did not show any alteration in FRET signal within this time frame (Figure 3A) although massive apoptotic morphological changes were evident (not shown).

Western blot analysis, using anti-GFP antibodies, of extracts of SH-SY5Y cells expressing the caspase-3 (DEVD) or control (LEVA) FRET sensors displayed a specific band with an apparent molecular weight of 130 kDa (Figure 3B). After 3 hours of incubation with 10 μM staurosporine a specific band at approximately 100 kDa appeared in cells expressing the caspase-3 (DEVD) sensor. EYFP and ECFP both have an expected size of 27 kDa, whereas the 441 amino acid long tau isoform in human brain tissue has been reported to migrate with an apparent molecular weight of approximately 74 kDa [11]. Thus, the ~100 kDa cleavage product is consistent with the expected migration of the EYFP-Tau fragment (~27 kDa + ~74 kDa) from caspase-dependent cleavage of the DEVD motif. In support of this is the fact that the ~100 kDa band was absent from staurosporine treated cells expressing the control (LEVA) sensor. In addition, the presence of caspase inhibitors prevented staurosporine-induced appearance of the ~100 kDa band (Additional file 1, Figure S1). The results demonstrate that decreased FRET intensity in apoptotic SH-SY5Y cells expressing the FRET sensor was correlated to caspase-dependent proteolysis of the sensor molecule.

### Detection of local caspase activation in live neuroblastoma cells

**Figure 4:**
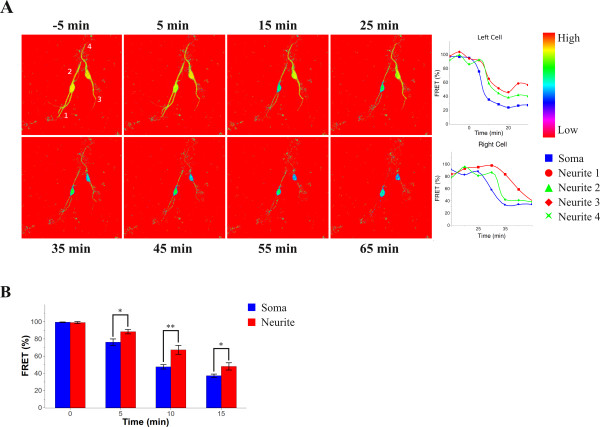
**Spatiotemporal differences in activation of caspase-6 in staurosporine treated neuroblastoma cells**. Differentiated SH-SY5Y cells expressing the caspase-6 (VEID) sensor molecule treated with 10 μM staurosporine were monitored by FRET time-lapse microscopy. (**A**) Images at 10 min intervals from a FRET time-lapse experiment. Time points relative to the FRET intensity change of the left cell are indicated at the top. The frame before the initial drop in FRET intensity was chosen as zero time point. The relative FRET intensity is shown in graphs corresponding to the left and right cell, respectively. The different graphs are from cell soma (blue squares); neurite 1 (red dots); neurite 2 (green triangles); neurite 3 (red rhombuses); neurite 4 (green crosses). Note the localized loss of FRET signal in the soma, which appeared earlier than in neurites. (**B**) Statistical analysis of spatiotemporal differences in FRET intensity of cell bodies (left, blue) and neurites (right, red) from different experiments (n = 8; mean ± S.E.M.). The FRET signals in cell bodies were significantly lower than the FRET signals in neurites at 5 min (p < 0.05), 10 min (p < 0.01) and 15 min (p < 0.05).

**Figure 5:**
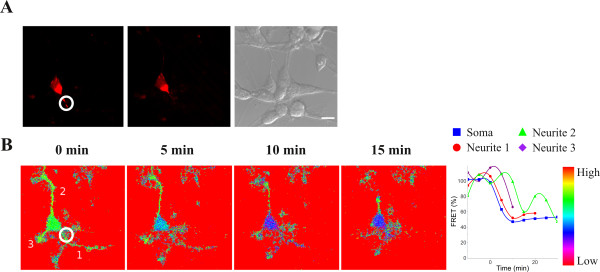
**Detection of caspase activity induced by local photo activation of mitochondrial KillerRed**. Differentiated neuroblastoma cells coexpressing mitochondrially targeted KillerRed and the caspase-3 (DEVD) sensor molecule. (**A**) Images of mitochondrial KillerRed fluorescence, with corresponding phase contrast image, before (circled) and after photo-activation in selected mitochondria. (**B**) Corresponding FRET intensity time-lapse images. Time points (see Figure 3) relative to the FRET intensity change are indicated at the top. The frame before the initial drop in FRET intensity was chosen as zero time point. The graph displays relative FRET intensity in the cell body (blue squares), neurite 1 (red dots), neurite 2 (green triangles) and neurite 3 (magenta rhombuses).

When we monitored the caspase activity in differentiated neuroblastoma cells expressing the caspase-6 (VEID) sensor treated with staurosporine we observed that the FRET intensity first decreased in the cell body and only 5-10 min later decreased in the neurites (Figure 4A and Additional file 2, Movie). Statistical analysis of a larger data set showed that the spatiotemporal difference was significant (Figure 4B). The staurosporine-induced decrease in FRET signal was significantly larger in cell bodies as compared to neurites at 5 min (p < 0,05), 10 min (p < 0.01) and 15 min (p < 0.05). This demonstrates the ability of the sensor molecule to monitor spatiotemporal changes in caspase activation in live cells in a reproducible way. Next, we decided to determine whether local caspase activation could also be detected using other sensors. For this, local damage was induced in differentiated SH-SY5Y cells co-expressing the caspase-3 (DEVD) sensor and the mitochondrially-targeted photosensitizer KillerRed [12]. Illumination of selected KillerRed containing mitochondria with 561 nm light made it possible to initiate apoptosis locally via the intrinsic pathway resulting in a localized loss of FRET signal (Figure 5). After local stimulation of selected mitochondria in a part of a neurite proximal to the cell body the intensity of the FRET signal from the caspase-3 (DEVD) sensor decreased throughout the targeted neurite and in the cell body, but only later decreased in neurites extending from other parts of the cell body. Thus, these results show that it is possible to visualize spatiotemporal differences in caspase activation in different parts of the cell using our FRET sensors.

### Detection of differential caspase activation in live neuroblastoma cells treated with Aβ_1-42 _peptide

**Figure 6:**
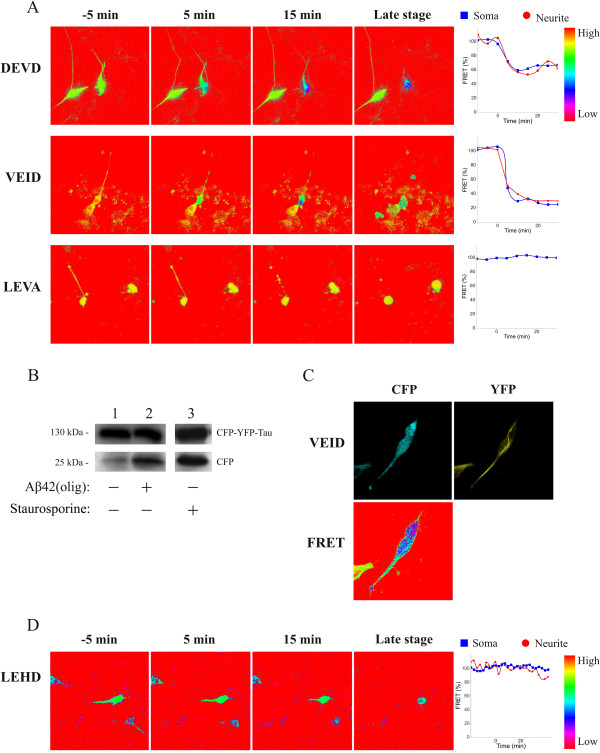
**FRET based monitoring of caspase activity in differentiated SH-SY5Y cells exposed to Aβ_1-42_**. Differentiated neuroblastoma cells expressing FRET sensor molecules were treated with oligomer-enriched preparations of Aβ_1-42 _at concentrations known to induce apoptosis. (**A**) FRET intensity time-lapse imaging of the caspase-3 and -6 sensors in cells treated with 20 μM oligomer-enriched Aβ_1-42_. Time is indicated on top. The frame before the initial drop in FRET intensity was chosen as zero time point for the caspase-3 (DEVD) and -6 (VEID) sensors, whereas initiation of neurite retraction was chosen as zero time point for the control (LEVA) sensor. Corresponding graphs show the quantitative change in FRET intensity in the soma (blue squares) and neurites (red dots). (**B**) Western blot analysis using anti-GFP antibodies, of FRET sensor molecules in extracts of cells expressing the caspase-6 (VEID) sensor incubated for 18 hours in the absence (lane 1) or presence (lane 2) of 10 μM of oligomer-enriched Aβ_1-42 _preparation. Staurosporine treatment (2 μM, 18 h) is shown for comparison. (**C**) Image of cell expressing the caspase-6 (VEID) sensor treated with oligomer-enriched Aβ_1-42 _showing loss of FRET signal, but maintained distribution of EYFP fluorescence along microtubules. ECFP fluorescence was diffuse. (**D**) FRET intensity time-lapse imaging of the caspase-9 (LEHD) sensor and corresponding quantitative graphic representation. Note the maintained high FRET intensity before and after neurite retraction, which was chosen as zero time point.

Next, we investigated if it was possible to monitor specific caspase activation induced by Aβ1-42 and to determine where in the cell this apoptotic signal is initiated. Differentiated SH-SY5Y cells expressing FRET sensor molecules for different caspases were exposed to a preparation enriched in Aβ1-42 oligomers (Additional file 3, Figure S2). Decreased FRET intensities for the caspase-3 (DEVD) and caspase-6 (VEID) sensors were observed (Figure 6A), that was accompanied by proteolysis as evidenced by a corresponding fragmentation of the caspase-6 (VEID) sensor (Figure 6B). As shown by the maintained localization of EYFP fluorescence to microtubules after the decrease in FRET intensity (Figure 6C), Aβ1-42 induced cleavage of the engineered VEID sequence ahead of any possible proteolysis that might affect localization. Even though we have shown that the FRET sensors enable detection of local changes (see Figure 4 and 5) the caspase-3 (DEVD) and caspase-6 (VEID) sensors displayed decreased FRET intensity throughout the cell. Thus, our results suggest that the Aβ1-42 peptide induces global activation of caspase-3 and caspase-6 in these cells. Interestingly, the FRET intensity from the caspase-9 (LEHD) sensor remained high even after morphological signs of cell death including neurite retraction (Figure 6D). This suggests that neurodegeneration induced by exposure to non-fibrillar forms of Aβ1-42 is not dependent on caspase-9 and that different caspase activities clearly can be distinguished by our sensors.

## Discussion

Selective elimination (degeneration or retraction) of axons and dendrites, without death of the neuron is important during development of the nervous system in order to refine synaptic connections. However, axon elimination, as well as apoptosis [13,14], also occurs in many neurological diseases or as a consequence of neuronal injury (reviewed by [1,15]). Axonal degeneration involves a rapid breakdown of the axonal cytoskeleton caused by enzymatic degradation of neurofilaments. A process termed dying-back degeneration (or Wallerian-like degeneration) has been described in a variety of neurological disorders and is characterized by initial degeneration of distal regions of long axons followed by distal-to-proximal progression. In many neurodegenerative diseases, including AD, axonal and dendritic atrophies are observed, (*e.g. *[16]). Such neuritic atrophies will most likely contribute significantly to the clinical symptoms and occur prior to or in the absence of neuronal death. In a mouse model of AD *in vivo *imaging studies indicated that the Aβ deposits, typically observed in AD brain, can indeed induce breakage of axonal and dendritic branches [17].

Here, we describe the development and characterization of new FRET based sensors for spatiotemporal imaging and analysis of caspase activation in relation to neurite degeneration and neuronal cell death. A full-length tau moiety was added to the FRET sensors both to mediate association to microtubules in neurites and thus increase signal intensity as well as to restrict diffusion of the sensors to allow monitoring progression of the apoptotic signal in a spatiotemporal manner (*cf*. Figure 1). As expected our caspase sensors localize to the cytoskeleton when expressed in non-neuronal (HeLa) cells and also to the microtubule of the neurites when expressed in differentiated neuronal cells. FRET studies in differentiated SH-SY5Y cells exposed to staurosporine verify the sensors as early markers of apoptosis occurring prior to neurite retraction and any other morphological changes could be detected. Caspase activation was measured as loss of FRET and it was confirmed, by western blot analysis of sensor fragmentation, to correspond to cleavage between EYFP and ECFP where the caspase cleavage sites are localized.

In this study we focus on three different caspases; caspase-3, -6, and -9. Caspase-9 is the initiator caspase associated with the mitochondrial or intrinsic apoptotic pathway [18]. Proapoptotic signals stimulating release of cytochrome c from mitochondria that promotes oligomerization of apoptotic peptidase activating factor 1 (Apaf-1), will lead to recruitment and activation of caspase-9. Caspase-9 null mice die perinatally and show a similar phenotype as mice with deletion of Apaf-1 or caspase-3 genes, including enlarged and deformed brains [19,20]. This clearly indicates the importance of the intrinsic apoptotic pathway during development of the nervous system. Caspase-9 has also been proposed to be activated by an alternative pathway: It has been shown that endoplasmic reticulum (ER) stress can activate caspase-9 in the absence of cytochrome c and Apaf-1 [21,22]. Interestingly, Aβ peptides have been shown to induce apoptosis mediated by ER stress [23]. Furthermore, increased levels of active caspase-9 have been detected in AD brain [15,24]. Also, in a recently published study [25] caspase-9 and the intrinsic apoptotic pathway was suggested to be involved in non-apoptotic caspase-3 activity in dendritic spines in Tg2576-APPswe mice. This activity correlated with the onset of memory deficits in these mice. Therefore, caspase-9 has been proposed to be involved not only in developmental apoptosis of neuronal cells but also in neurodegenerative disorders such as AD. Caspase-3 is the major executioner caspase and can be activated once an initiator caspase, like caspase-9, is activated. Caspase-6 is also often classified as an executioner caspase since it cleaves the nuclear lamin proteins during apoptosis [6]. However, proteomics analysis showed that nearly half of identified neuronal caspase-6 targets were cytoskeleton-associated, indicating that caspase-6 most likely can function also before death signals reach the nucleus [26]. In addition, recent studies have implicated caspase-6 in axonal degeneration not linked to apoptotic cell death [3].

We evaluated the performance of the caspase sensors in differentiated neuroblastoma cells treated with staurosporine. We observed a significant decrease in FRET signal in cells expressing caspase-3 and -9 sensors, but not in cells expressing the non-cleavable control (Figure 3). Staurosporine is a non-specific inhibitor of protein kinases and is one of the best inducers of apoptosis in different types of cells, including neuroblastoma [27,28]. Although the mechanism by which staurosporine induces apoptosis is not well known, the mitochondrial pathway has been shown to be involved [29,30], consistent with our results. For caspase-3 and -9, we could not detect any time difference in the loss of FRET in neurites and soma. This indicates that staurosporine may induce a global activation of these two caspases in neuronal cells. However, when the loss of FRET in cells expressing the caspase-6 sensor was analyzed we observed a clear and significant delay in the neurites compared to the cell bodies (Figure 4). This indicates a difference in staurosporine-induced apoptotic signaling in axons/dendrites compared to the cell bodies. A previous study has indicated a spatiotemporal difference in caspase-3 compared to caspase-6 activation [31]. In undifferentiated SH-SY5Y cells exposed to staurosporine caspase-6 activation, monitored by the appearance of a lamin B1 fragment in western blot analysis, was considerably delayed compared to cleavage of caspase-3 substrates [31]. Thus, it may be speculated that staurosporine induces a delayed activation of caspase-6 that is initiated in the cell body and thereafter is spread to the neurites.

The ability to detect caspase activation at sub-cellular resolution was further established using a co-expressed mitochondrial photosensitizer (Killer red) [12], which enabled induction of apoptosis locally in the cell via the intrinsic pathway. We observed that if caspase-3 activation was initiated in one of the neurites the signal was quickly transferred to the cell body but not to other neurites (Figure 5). The other neurites degenerated as well after the cell had been exposed to the death stimuli in spite of the delayed caspase-3 activation.

Finally, we used differentiated neuroblastoma cells expressing caspase sensors to analyze the effect of the Aβ peptide on apoptotic signaling. Apoptotic signaling is believed to play an important role in a number of neurological disorders, including AD [32]. If caspase activation is crucial for the progression of AD pathology it is important to learn how the activation occurs, where in the neuron this process takes place, and whether or not it will result in neuronal cell death. Aβ is a hydrophobic peptide generated by sequential proteolytic cleavage of amyloid precursor protein (APP) by β- and γ-secretase and is the leading candidate for activation of apoptotic mechanisms in AD [7,33]. Aβ is highly prone to aggregate and in the brain it is found in different forms ranging from monomers and non-fibrillar aggregates, termed oligomers, to a highly fibrillar form. Recent evidences suggest that it is the diffusible Aβ oligomers that have the most toxic properties [34,35]. Aβ oligomers have also been shown to bind to synaptic sites [36], which may be important for early synaptic failure observed in AD (*cf. *[37]). Furthermore, Aβ has been proposed to have a local effect on neurites causing degeneration and neuritic apoptosis using compartmented cultures of hippocampal neurons [38]. In the present study we have exposed the cells to a preparation enriched in oligomer-enriched forms of Aβ with non-detectable levels of fibrils. Aβ exposure resulted in activation of caspase-3 and -6, but we could not detect any specific spatiotemporal effect of the peptide. Thus, our results suggest that in these neuronally differentiated cells there is no part of the cell that is more vulnerable to insult and that Aβ rather induces global caspase activation. Because the caspase-9 sensor resisted proteolysis, the intrinsic pathway may play a less important role in Aβ_1-42 _induced apoptosis of these cells. Most interestingly, it was recently shown in a mouse model that caspase activation precedes and leads to tangles (*i.e*., a characteristic neuropathological feature in AD). Evidences also indicated that caspase-3 and -6 were activated in the tangle positive cells [39].

In conclusion, we show that the anchored caspase sensors localize to the cytoskeleton, that they are differentially cleaved depending on stimuli and that they can be used to detect local caspase activation and to follow the spreading of caspase activation from one part of the cell to another. Under the conditions used in this study we could not detect any specific spatiotemporal effect of Aβ on caspase activation. Caspase-3 and -6, but not -9, were activated both in soma and in neurites. It will be interesting to use the anchored FRET sensors in experiments designed to cause retraction and loss of synaptic contacts without causing cell death. This is indeed a promising new tool in research on AD and other neurodegenerative conditions.

## Materials and methods

### Gene construction

To construct the localized caspase sensors, EYFP was amplified from the EYFP-C1 plasmid (BD Biosciences) using the following primers: forward, 5'-TATTAGATCTCATGGTGAGCAAGGGCGA-G-3', reverse, 5'-ACGTGAATTCCTTGTACAGCTCGTCCATGC-3'. The resulting PCR product was digested with *Bgl*II-*Eco*RI, gel-purified and ligated into the *Bgl*II-*Eco*RI of the ECFP-C1 vector (BD Biosciences). The longest isoform of human tau (441 amino acids) was amplified from the pBThtau40 plasmid (kindly provided by Dr. Jacek Biernat, Max-Planck-Gesellschaft) using the following primers: forward, 5'-ATTAGAATTCATGGCTGAGCCCCGCCAG-3', reverse, 5'-ATGCGGATCCGTGATCACAAAC-3'. The resulting PCR product was digested with *Eco*RI-*Bam*HI, gel-purified and ligated into the *Eco*RI-*Bam*HI of the ECFP-EYFP vector. To create cleavage sites for caspase-3, -6 and -9 plus an un-cleavable control sequence single stranded sense 32-mers; 5'-CCGGAGCAGATGAGGTCGATGCCGATGAGGTC-3' (caspase-3, DEVDx2), 5'-CCGGAGCAGTCGAGATAGATGCCGTAGAGATC-3' (caspase-6, VEIDx2), 5'-CCGGAGCACTGGAGCACGATGCCCTGGAGCAC-3' (caspase-9, LEHDx2) and 5'-CCGGAGCACTGGAGGTCGCCCTGGAGGTCGCC-3' (control, LEVAx2) were annealed to complementary anti-sense 32-mers; 5'-GATCGACCTCATCGGCATCGACCTCATCTGCT-3' (caspase-3), 5'-GATCGATCTCTACGGCATCTATCTCGACTGCT-3' (caspase-6), 5'-GATCGTGCTCCAGGGCATCGTGCTCCAGTGCT-3' (caspase-9) and 5'-GATCGGCGACCTCCAGGGCGACCTCCAGTGCT-3' (control) generating double stranded DNA with caspase cleavage sites in tandem and *Bsp*EI-*Bgl*II sticky ends that were ligated into the *Bsp*EI-*Bgl*II of the ECFP-EYPP-Tau vector. One base was changed in the *Bgl*II site in the linker region to facilitate restriction analysis.

### Cell culture and transfection

HeLa (CCL-2, American Type culture Collection) and U2OS cells (ATCC-HTB-96, LGC standards) were cultured in Dulbecco's Modified Eagle Medium (DMEM, Invitrogen) supplemented with 10% fetal bovine serum (FBS, Invitrogen), 100 U/ml penicillin and 100 μg/ml streptomycin (PEST, Invitrogen). Human neuroblastoma SH-SY5Y cells (CRL-2266, ATCC) were cultured in DMEM/F12 supplemented with 10% FBS, 100 U/ml PEST. The cells were maintained in a humidified 5% CO_2 _atmosphere at 37°C. Cells were transfected using FuGENE HD (Roche). In live cell imaging experiments cells were seeded on to glass cover slips prior to differentiation. The neuroblastoma cells were differentiated with 10 μM retinoic acid (RA) for three days before transfection after witch cells were incubated for one day prior to the experiment. For western blot analysis cells were seeded at a density of 25.000 cells/cm^2 ^into Petri dishes (Nunc, Denmark). The following day the cells were treated with 10 μM RA for 72 h. After 24 h, the cells were transfected with plasmids encoding different caspase sensors and incubated for 48 h prior to the experiment.

### Preparation of Aβ1-42 enriched in oligomers

Aβ1-42 (American peptide) was dissolved in hexafluoroisopropanol (HFP) to remove any preexisting structures in the lyophilized stocks and was then stored at -70°C. The peptide was prepared to obtain a solution enriched in oligomeric, but devoid of fibrillar, forms of the peptide, cf. [40]. The oligomer-enriched Aβ1-42 was prepared freshly before each experiment. HFP was allowed to evaporate from the tube using speedvac system for 5 min at room temperature. Thereafter, Aβ1-42 was dissolved in dimethyl sulfoxide (DMSO) to a concentration of 5 mM. Subsequently, phosphate bovine saline (PBS) was added to give a final Aβ1-42 concentration of 100 μM. Finally, the preparation was vigorously vortexed at room temperature for 10 min, whereafter Aβ1-42 was allowed to aggregate by incubation for 24 h at 4°C at 200 rpm. Analysis of the Aβ preparation was made by western blot and Thioflavin T assay (Additional file 2, Figure S2).

### Imaging

Imaging was performed on a Leica TCS-SP laser scanning confocal microscope (Leica, Heidelberg, Germany) with a 63.0 × 1.32 oil immersion objective using a 442 nm 10 mW HeCd laser, 488 nm laser line of a 40 mW Ar-laser and 561 nm laser line of a yellow diode pumped solid-state 50 mW laser. Emission was collected between 455-505 nm (CFP), 515-575 nm (YFP and FRET), and 580-650 nm (KillerRed) using a long pass dicroic mirror (LP) 450 for CFP and FRET, LP500 for YFP and triple dicroic 488/561/633 for KillerRed. The laser lines were scanned sequentially to minimize bleed through. Induction of KillerRed phototoxic effect was achieved by scanning a region of the cells using the 561 nm laser line. Live cell imaging was performed by mounting cells in sealed chambers with serum free media buffered with HEPES. In experiments with staurosporine and Aβ, the agent was added to the media when mounting the cells. Temperature was maintained at 37°C using the Cube and Box microscope incubator system (Live Imaging Services). Projections of image stacks were used for quantification. Images were processed and quantified using ImageJ (Rasband, W.S., Image J, U. S. National Institutes of Health, Bethesda, Maryland, USA, http://rsb.info.nih.gov/ij/, 1997-2009). To acquire the ratio-metric images the intensity in the FRET channel was divided by the intensity in the CFP channel. A pseudo-color was applied and adjusted to visualize the changes in FRET. For graphic representation, images were segmented in a semi-automatic process rendering soma and neurite regions of interest. Values were normalized in relation to the mean value prior to changes in FRET. The last frame before decreased FRET could be detected in each individual experiment was chosen as zero time point to enable alignment of graphs from different experiments. At least three individual experiments were performed. Spatiotemporal studies of FRET signals in neurites and soma were statistically analyzed using unpaired two-tailed Student' s T-test.

### Western blot analysis

For western blot analysis of caspase sensor fragmentation human neuroblastoma SH-SY5Y cells treated with Aβ1-42 or staurosporine were harvested as follows: First, the cells were washed twice with PBS containing 0.7 μg/ml aprotinin (from bovine) and 0.8 mM PMSF. Thereafter, the cells were lysed in lysis buffer (150 mM NaCl, 50 mM TrisCl, 1 mM EDTA, 1% NP-40), containing complete protease inhibitor cocktail, for 20 min at 4°C. In order to remove cell debris the lysate was centrifuged at 10 000 g for 5 min at 4°C. The supernatant was then used for western blot analysis. 30 μg protein from each separate sample was separated by electrophoresis on 8% SDS-polyacrylamide gels and subsequently transferred to polyvinyldine difluoride membranes at 350 mA for 3 h. The membrane was blocked in PBS, containing 5% dry milk (w/v) and 0.1% Tween, for 1.5 h, followed by over night incubation at 4°C with primary rabbit polyclonal or mouse monoclonal anti-GFP antibody (1:1000) (R&D systems). Finally, secondary horseradish peroxidise-coupled donkey anti-rabbit or sheep anti-mouse antibodies (1:5000) were incubated for 1 h at room temperature and the detected proteins were visualised by chemiluminescence using Western LightingTM-PLUS-ECL kit (Perkin Elmer, Netherlands). All western blot reagents were from GE Healthcare or Bio-Rad Laboratories.

## Competing interests

The authors declare that they have no competing interests.

## Authors' contributions

The original design of this study was made by RAF, VR, KI and EH. cDNA construction was made by VR, MZ and MS. Cell culture, transfection, treatments and microscopy was performed by RAF, VR and TG and image analysis was performed by RAF. TG performed western blot analysis. RAF and VR coordinated the experiments. RAF, VR, TG, KI and EH wrote the manuscript. All authors have read and approved the final version of the manuscript.

## Supplementary Material

Additional file 1 **Figure S1 Inhibition of staurosporine-induced FRET-sensor cleavage by caspase inhibitors**. western blot analysis using anti-GFP antibodies of FRET sensor molecules in cells expressing the VEID sensor in the presence or absence (1 h pretreatment) of the caspase inhibitors Z-VAD-FMK (Promega) or Z-Asp-CH2-DCB (PeptaNova) followed by 1 h staurosporine treatment. Click here for file

Additional file 2 **Movie. Spatiotemporal difference in activation of caspase-6 in staurosporine treated neuroblastoma cells**. Differentiated SH-SY5Y cells expressing the caspase-6 (VEID) sensor molecule treated with staurosporine were monitored by FRET time-lapse microscopy. One frame every 5 min. Click here for file

Additional file 3 **Figure S2 Analysis of oligomer- and fibril-enriched preparations of Aβ_1-42_**. **(A**) Representative western blot showing high levels of fibrilar Aβ in the oligomeric/fibrillar preparation (lane 1) and the absence of Aβ fibrils in the oligomeric preparation (lane 2). The Aβ samples were separated by SDS-PAGE and both stacking- and separation gel was analyzed by western blot using 6E10 antibodies (Signet Laboratories). (**B**) Statistical analysis of Thioflavin T (T3516, Sigma) fluorescence in oligomeric/fibril-enriched Aβ preparations (fAβ; open bar) and oligomer-enriched Aβ preparations (oAβ; filled bar). Aβ samples were analyzed in phenol red-free medium and according to the manufacturer's instructions. The data represent mean ± SEM for three independent experiments. A.U., arbitrary units. *p < 0.05 significantly different from fAβ. Click here for file
