# Supplementary figures and images for: Anchored FRET sensors detect local caspase activation prior to neuronal degeneration
Source: Mol Neurodegener. 2011 May 23;6:35. doi: 10.1186/1750-1326-6-35 (PMC3121597; doi:10.1186/1750-1326-6-35)

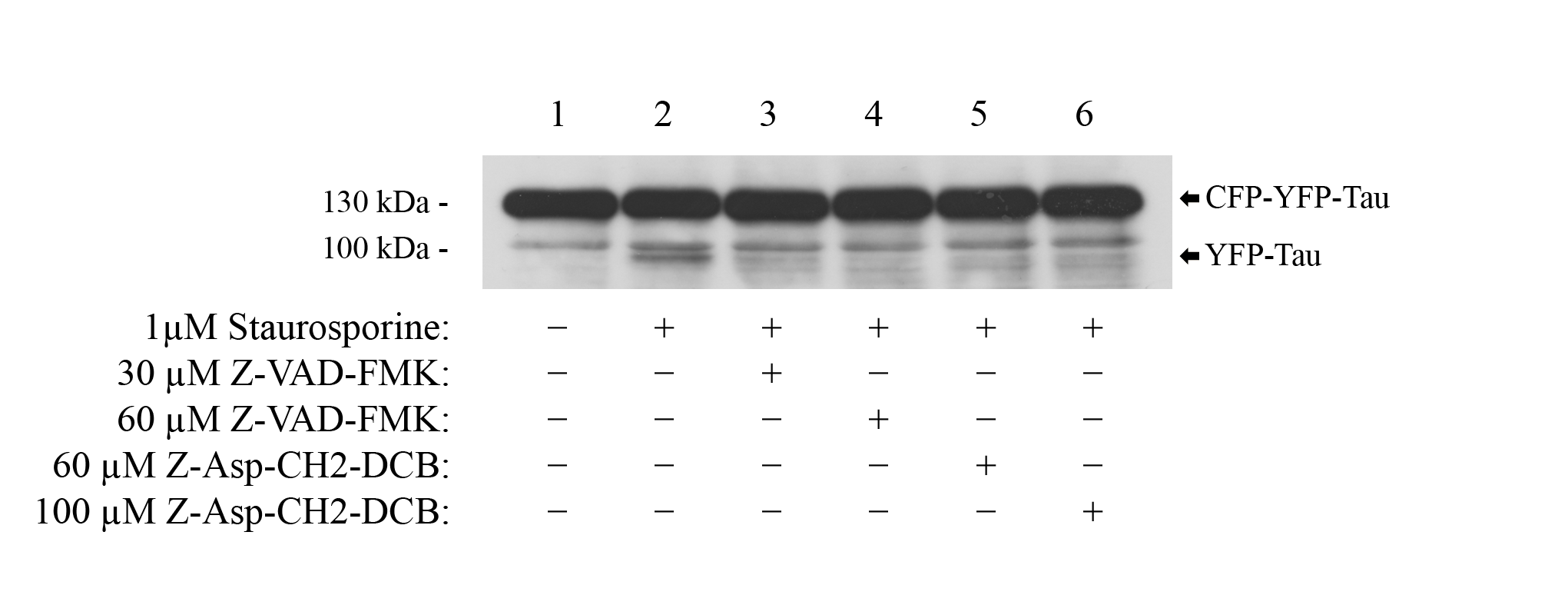

Supplement: Additional file 1 — Figure S1 Inhibition of staurosporine-induced FRET-sensor cleavage by caspase inhibitors. western blot analysis using anti-GFP antibodies of FRET sensor molecules in cells expressing the VEID sensor in the presence or absence (1 h pretreatment) of the caspase inhibitors Z-VAD-FMK (Promega) or Z-Asp-CH2-DCB (PeptaNova) followed by 1 h staurosporine treatment. [file 1750-1326-6-35-S1.PNG]

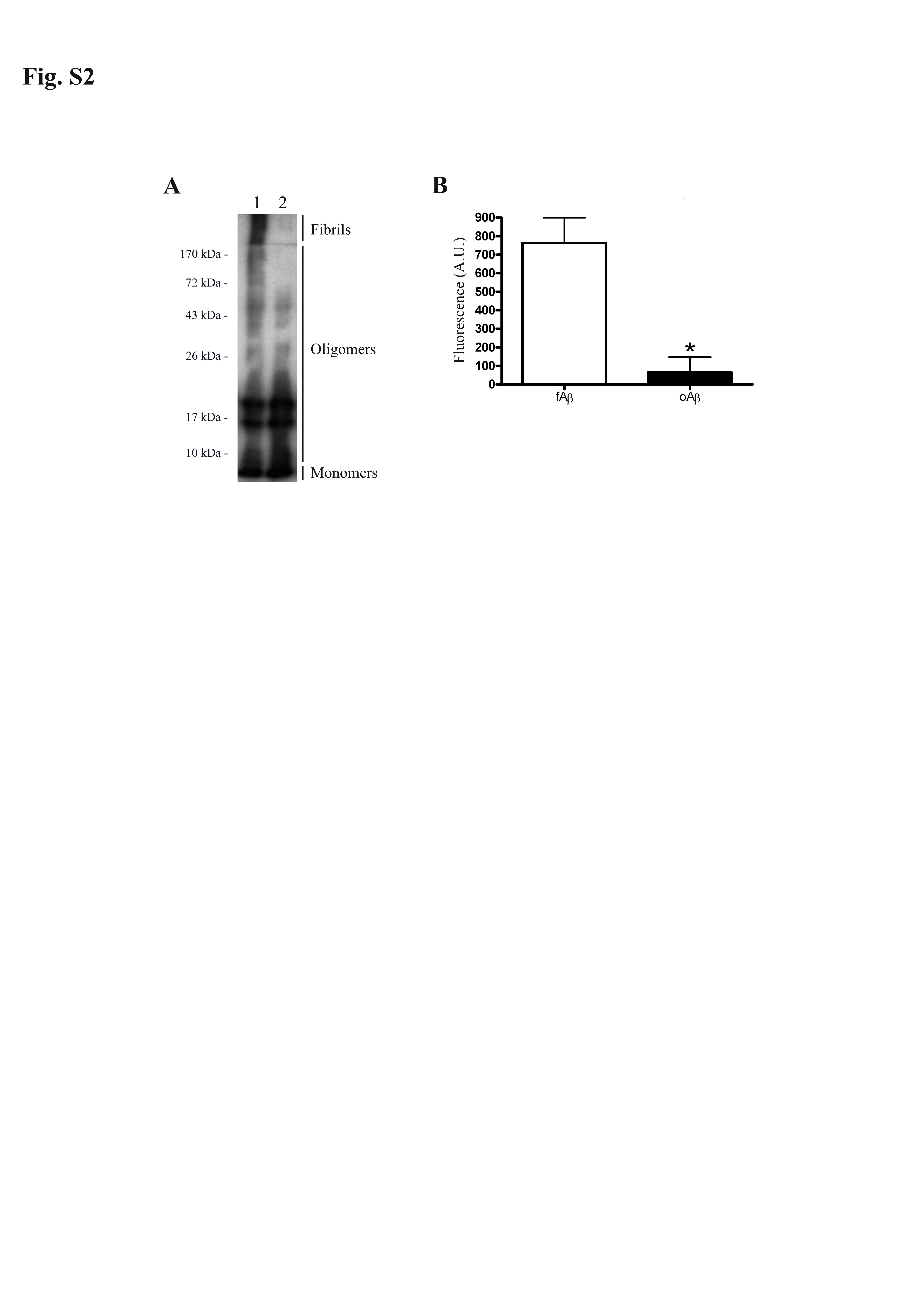

Supplement: Additional file 3 — Figure S2 Analysis of oligomer- and fibril-enriched preparations of Aβ1-42. (A) Representative western blot showing high levels of fibrilar Aβ in the oligomeric/fibrillar preparation (lane 1) and the absence of Aβ fibrils in the oligomeric preparation (lane 2). The Aβ samples were separated by SDS-PAGE and both stacking- and separation gel was analyzed by western blot using 6E10 antibodies (Signet Laboratories). (B) Statistical analysis of Thioflavin T (T3516, Sigma) fluorescence in oligomeric/fibril-enriched Aβ preparations (fAβ; open bar) and oligomer-enriched Aβ preparations (oAβ; filled bar). Aβ samples were analyzed in phenol red-free medium and according to the manufacturer's instructions. The data represent mean ± SEM for three independent experiments. A.U., arbitrary units. *p < 0.05 significantly different from fAβ. [file 1750-1326-6-35-S3.JPEG]
